# Supplementary material for: The interactome of intact mitochondria by cross-linking mass spectrometry provides evidence for coexisting respiratory supercomplexes
Source: Mol Cell Proteomics. 2017 Dec 8;17(2):216–32. doi: 10.1074/mcp.RA117.000470 (PMC5795388; doi:10.1074/mcp.RA117.000470)
Supplement: Supplemental Data [file supp_RA117.000470_133922_0_supp_23978_fzffwf.zip › spectra_annotation/mito_DR_spectra_annotation/206-1-2-1-6-1.pdf]

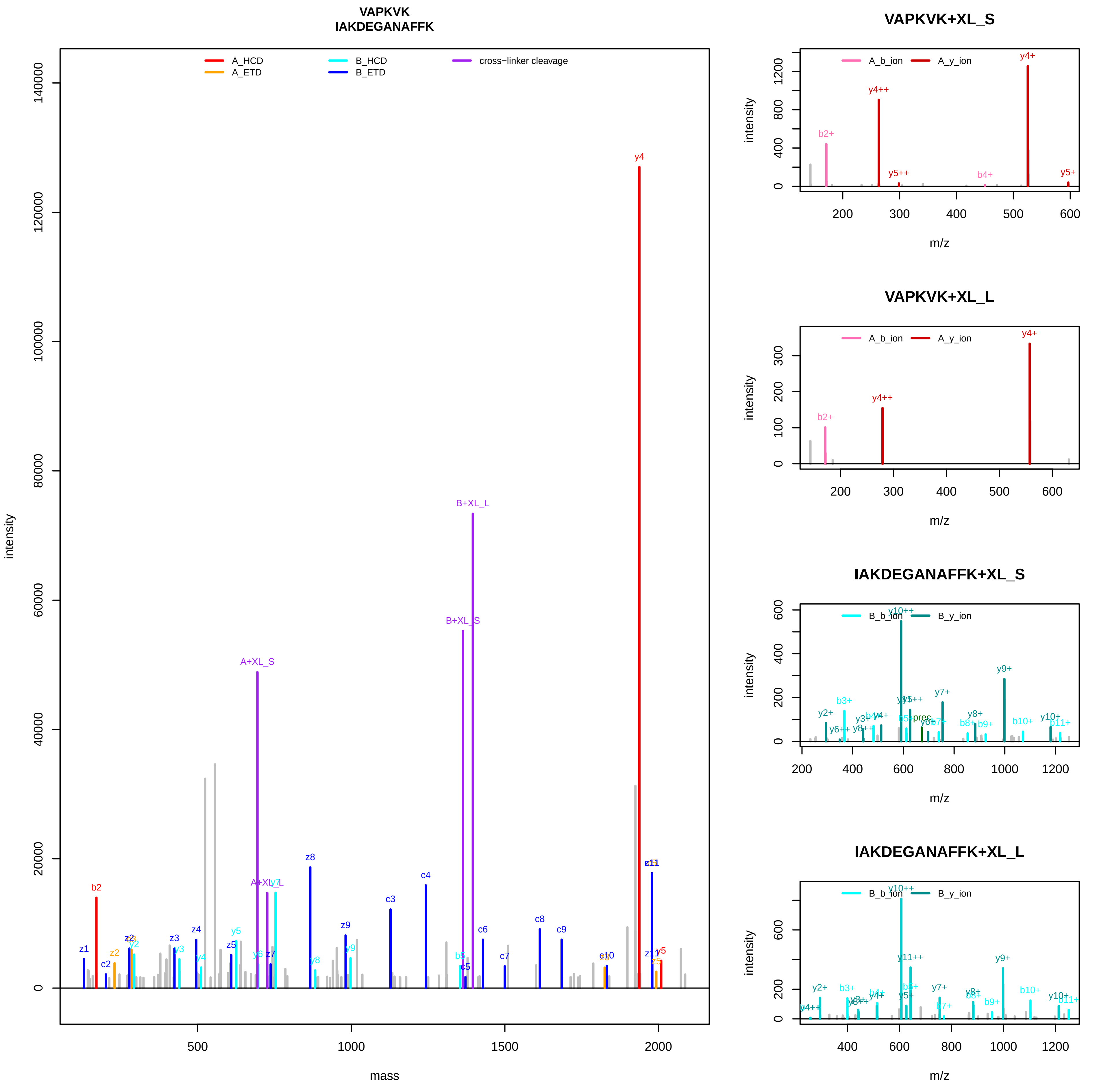

VAPKVK
IAKDEGANAFFK
A_HCD
A_ETD
B_HCD
B_ETD
cross−linker cleavage
intensity
mass
0
20000
40000
60000
80000
100000
120000
140000
500
1000
1500
2000
y4
B+XL_L
B+XL_S
A+XL_S
A+XL_L
b2
z1
c2
z2
y2
z3
y3
z4
y4
y5
z5
y6
z7
y7
z8
y8
z9
y9
c3
c4
b5
c5
c6
c7
c8
c9
c10
z11
y5
VAPKVK+XL_S
A_b_ion
A_y_ion
y4+
y4++
b2+
y5++
b4+
y5+
intensity
m/z
0
400
800
1200
200
300
400
500
600
VAPKVK+XL_L
A_b_ion
A_y_ion
y4+
y4++
b2+
intensity
m/z
0
100
200
300
200
300
400
500
600
IAKDEGANAFFK+XL_S
B_b_ion
B_y_ion
y10++
y9+
y7+
b3+
y2+
y8+
b10+
y10+
b11+
intensity
m/z
0
200
400
600
200
400
600
800
1000
1200
IAKDEGANAFFK+XL_L
B_b_ion
B_y_ion
y10++
y11++
y9+
y2+
b3+
y7+
b10+
y10+
b11+
intensity
m/z
0
200
600
400
600
800
1000
1200
